# Effects of intraoperative magnesium sulfate administration on postoperative tramadol requirement in liver transplant patients

**DOI:** 10.1186/2197-425X-3-S1-A694

**Published:** 2015-10-01

**Authors:** B Gucyetmez, HK Atalan, S Aslan, M Berktas, S Yazar, A Erturer, IR Sozenoglu, TB Denizalti, KY Polat

**Affiliations:** Intensive Care Unit, Acibadem International Hospital, Istanbul, Turkey; Intensive Care Unit, Atasehir Memorial Hospital, Istanbul, Turkey; Department of Transplantation, Atasehir Memorial Hospital, Istanbul, Turkey; Yeditepe University, PEPIRC, Istanbul, Turkey

## Introduction

Magnesium is an N-methyl-D-aspartate receptor blocker and it's known to have analgesic effect([1, 2]). Hypomagnesaemia is often seen in major surgery and it is associated with higher morbidity, mortality, organ dysfunction, systemic inflammatory response syndrome and pulmonary hypertension([2, 4]). It's shown that intra-operative use of magnesium sulfate reduced per-operative analgesic requirement([5]).

## Objectives

The purpose of the present study is to investigate the effects of intraoperative magnesium sulfate administration on postoperative tramadol requirement in liver transplant patients.

## Methods

Upon the approval of local ethical committee, liver transplant patients >18 years were screened prospectively between October 2014 and April 2015. All patients were received standart anesthesia induction (1,5 mcg/kg fentanyl, 2 mgr/kg propofol and 0,6 mg/kg esmeron) and maintenance (MAC>0.7 sevorane, 0,05-0,25 mcg/kg/min remifentanil and 0,15 mg/kg esmeron per hour). Of the screened ones; 35 randomly selected patients with normal blood magnesium level (1.8-3.6 mg/dL) were included to control group and another 35 randomly selected patients with low blood magnesium level

(< 1.8 mg/dL) were included to magnesium group and given 50 mg/kg intravenous magnesium sulfate replacement by the anaesthetist team. Intravenous tramadol (0,15 mgr/kg/h infusion and 0,2 mg/kg bolus if visuel pain scores >5) was used for postoperative analgesia for all patients. Patient's demographic datas, model for end-stage liver disease (MELD) scores, lenght of time for surgery, intra-operative magnesiun levels, APACHE II and SOFA Scores, 24-hours tramadol requirement, time of the first additional tramadol administration, mechanical ventilation (MV) duration, length of ICU and hospital stay were recorded by the intensivists.

## Results

Magnesium and control groups were similar in terms of demographics, MELD score, length of time for surgery, APACHE II score and length of ICU stay (p>0.05 for each). Median intraoperative magnesium level (1.7 mg/dL vs. 2.2 mg/dL), 24-hours tramadol requirement (3.73 mg/kg/day vs. 4.13 mg/kg/day) and MV duration (6.0 hours vs. 8.0 hours) of magnesium group were significantly lower than control group whereas median time of the first additional tramadol use (18.0 hours vs. 5.0 hours) was significantly higher (p < 0.001 for all).

## Conclusions

Intraoperative use of magnesium sulfate in the liver transplanlation patients reduces postoperative tramadol requirement and thus it is a candidate to be adjuvant agent with its advantages. Besides, it may reduce MV duration by contributing to effective analgesia without causing respiratory depression.
